# Oscillatory EEG Signatures of Affective Processes during Interaction with Adaptive Computer Systems

**DOI:** 10.3390/brainsci11010035

**Published:** 2020-12-31

**Authors:** Mathias Vukelić, Katharina Lingelbach, Kathrin Pollmann, Matthias Peissner

**Affiliations:** 1 Fraunhofer Institute for Industrial Engineering IAO, 70569 Stuttgart, Germany; kathrin.pollmann@iao.fraunhofer.de (K.P.); matthias.peissner@iao.fraunhofer.de (M.P.); 2 Institute of Human Factors and Technology Management IAT, University of Stuttgart, 70569 Stuttgart, Germany; katharina.lingelbach@iat.uni-stuttgart.de; 3 Department of Psychology, University of Oldenburg, 26129 Oldenburg, Germany

**Keywords:** affective reactions, human–computer interaction, adaptive assistance system, electroencephalography, event-related desynchronization, event-related synchronization, functional connectivity

## Abstract

Affect monitoring is being discussed as a novel strategy to make adaptive systems more user-oriented. Basic knowledge about oscillatory processes and functional connectivity underlying affect during naturalistic human–computer interactions (HCI) is, however, scarce. This study assessed local oscillatory power entrainment and distributed functional connectivity in a close-to-naturalistic HCI-paradigm. Sixteen participants interacted with a simulated assistance system which deliberately evoked positive (supporting goal-achievement) and negative (impeding goal-achievement) affective reactions. Electroencephalography (EEG) was used to examine the reactivity of the cortical system during the interaction by studying both event-related (de-)synchronization (ERD/ERS) and event-related functional coupling of cortical networks towards system-initiated assistance. Significantly higher α-band and β-band ERD in centro-parietal and parieto-occipital regions and β-band ERD in bi-lateral fronto-central regions were observed during impeding system behavior. Supportive system behavior activated significantly higher γ-band ERS in bi-hemispheric parietal-occipital regions. This was accompanied by functional coupling of remote β-band and γ-band activity in the medial frontal, left fronto-central and parietal regions, respectively. Our findings identify oscillatory signatures of positive and negative affective processes as reactions to system-initiated assistance. The findings contribute to the development of EEG-based neuroadaptive assistance loops by suggesting a non-obtrusive method for monitoring affect in HCI.

## 1. Introduction

In recent years, the technical capabilities and the widespread use of interactive computer systems have increased enormously. This has led to rising interest in the question of how we can improve human–computer interaction (HCI). While early HCI research has focused mainly on perceptual, cognitive and physical aspects, more and more attention is now directed towards affective processes related to the interaction between human and computer (e.g., [1]). In order to investigate affective user reactions during the HCI, subjective measures such as questionnaires and interviews are commonly used [2]. With the rise of more sophisticated mobile sensor technologies, HCI research started to explore the potential of using neurophysiological signals to capture affective user processes during their interaction with computers [3,4,5,6,7,8,9]. Neurophysiological measurements enable the continuous monitoring of affective processes without disturbing the user, concurrently avoiding possible memory and introspective biases. This knowledge can help to design interactive computer systems in a more user-oriented manner. Moreover, continuous neurophysiological measures of affect can enable neuroadaptive system loops to adapt the system’s behavior and assistance in real-time according to the current affective user state [10,11,12]. However, before such systems can be applied outside research labs and beyond medical scenarios, it is necessary to gain a reliable understanding of the brain processes underlying affective reactions in HCI.

While considerable progress has been made in understanding the principles of brain functions and organization that underlie affective processing [13,14], there is currently a gap between research in the fields of affective neuroscience and HCI. Most studies primarily use well-established and standardized stimulus material such as pictures, sounds or movies to investigate the brain mechanisms of affect [15,16,17,18,19]. However, those materials may lack ecological validity [20] to investigate affective user reactions in real-world HCI applications. This creates a need for more naturalistic and complex experimental environments as well as tasks with active integration of the participants. Moreover, it can be assumed that affect reactions during HCI will be significantly lower than the reactions to standardized stimuli that has been specifically selected to generate strong emotional reactions such as pictures of an injured person after a car crash or cute puppies [18]. These standardized stimuli cause immediate affective reactions in terms of approach or withdrawal [21,22].

There have been only a few attempts to capture the underlying neurophysiological signatures of affect during HCI. These studies were based on the idea that a technical system changes its visual appearance or behavior to induce certain affective states [5,6,7,23,24,25]. While this generally appears to be a valid approach to assess affect during HCI, it should be noted that these studies have primarily focused on negative affect. Moreover, some of the studies used gaming interaction scenarios [6,7,24,26]. Brain processes underlying positive affect during HCI and the extent to which positive and negative affect differs from each other have largely been neglected. Therefore, it is important to identify neurophysiological signatures of positive and negative affective processes during more naturalistic interactions. Furthermore, this might help to exploit the potential of real-time affect monitoring for neuroadaptive applications.

Functional magnetic resonance imaging (fMRI) has been used as a preferred measurement technique to unravel subcortical and cortical structures involved in affect processing [27,28,29]. However, due to its immobility and limited temporal resolution, this technique is not well suited for applied research in the context of HCI. Several recent studies highlighted the great potential of the electroencephalography (EEG) for capturing and recognizing affective processes using computational models and various machine learning algorithms [8,30,31,32,33,34]. EEG recordings provide insights in brain processes with a fine temporal scale up to milliseconds range. Furthermore, it is a portable and non-invasive technique allowing participants to maintain a rather comfortable position. EEG, therefore, offers a great ecological validity for research in HCI. Numerous studies have revealed the precise timing of affect with event-related potentials (ERPs) indexing different stages of affective stimulus processing and perception [35,36,37,38,39]. Beside ERP-analysis, EEG provides the possibility to study frequency-specific measures of oscillatory power ranging from lower frequencies such as the θ-band (4–7 Hz) up to higher frequencies in the γ-band (>35 Hz). Moreover, it allows the examination of measures of the inter-relation between certain cortical regions based on frequency-specific functional connectivity networks. Both power and functional connectivity measures have been related to affect highlighting correlations with valence, arousal and appraisal states [40,41,42,43,44,45,46,47,48,49,50,51,52,53,54]. However, to our knowledge, such correlates of affect have not been systematically investigated in the context of HCI and in more-naturalistic experimental environments.

The present study aims at promoting the development of EEG-based neuroadaptive systems by providing a comprehensive picture of the underlying oscillatory EEG signatures of positive and negative affect. The experimental task design and cover story (see Section 2.2 for more details) mirrored the one described by [55]. The chosen paradigm—AFFINDU—is especially suited for investigating correlates of affect during HCI because it (a) provides a non-game based, close to realistic HCI scenario and (b) can be used to induce affective states of different valence (positive and negative) [55]. The design of the adaptive behavior is based on the appraisal and the componential theory of affect [56,57,58]. Those theories state that an event that supports goal achievement is perceived as positive while an event that impedes goal achievement is experienced as negative. In a previous study [55], we evaluated to what extent the interaction behavior during navigation tasks are subjectively perceived as supportive and impeding. In addition, we investigated whether the designed events successfully induced the intended affective states in the participants. Furthermore, we were interested in the overall affective experience during the interaction with AFFINDU. Our results showed that AFFINDU is a valid experimental paradigm to induce positive and negative levels of affect. In this current study, we want to extend our previous research by analyzing the oscillatory EEG signatures of affective processes in a larger cohort of participants. We focus our investigations on the frequency power and functional connectivity responses to impeding and supportive behavior of the simulated adaptive system behavior (AFFINDU, described in the methods section) during a navigation task. Similarly to the conceptual framework of cognitive probing [59], AFFINDU allows us to capture the participant’s affective processes towards the adaptive system behavior during a navigation task. To our best knowledge, this study is the first attempt to investigate the EEG oscillatory underpinnings of affective processes during a generic navigation task. Hence, our study is designed as an exploratory study. We expect that the reactivity of the participants’ brains towards the system behavior would be reflected in different modulations of the frequency power and functional connectivity that showed correlations with valence, arousal and appraisal in previous studies [34,60,61].

## 2. Materials and Methods

### 2.1. Participants

Sixteen right-handed healthy volunteers ($M_{age}$ = 26.38, *SD* = 3.22 years, seven female) were recruited for the current study. Handedness was assessed by the Edinburgh Handedness Inventory. Participants gave their written informed consent before participation and received monetary compensation. The study protocol was approved by the local ethics committee of the Medical Faculty of the University of Tuebingen, Germany (1922015BO2).

### 2.2. General Study Design and Cover Story

**Figure 1:**
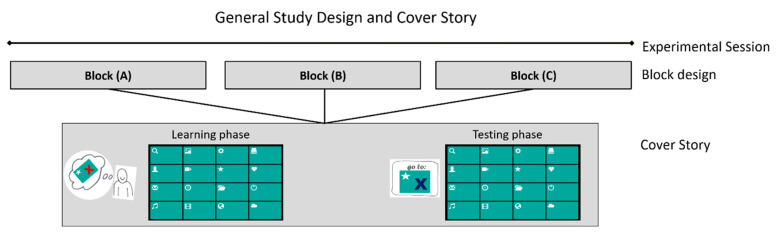
Schematic illustration of the experimental paradigm and cover story of AFFINDU used during the experimental session (for details please see the material and methods section).

**Figure 2:**
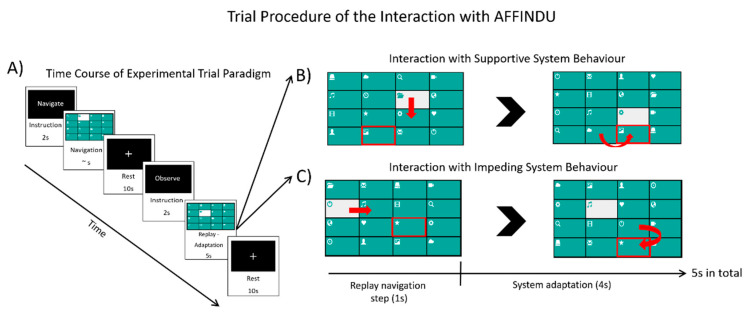
Schematic illustration of the trial procedure. (**A**) Trial based procedure of the experimental session. (**B**,**C**) The two different implemented system behaviors of AFFINDU, supportive and impeding, respectively.

The experimental paradigm AFFINDU was implemented as a grid-based menu with 4 × 4 items, through which the participants navigated in a controlled step-by-step sequence. Each item of the grid menu was labelled by a monochrome icon representing an application such as TV guide, weather forecast or emailing. The graphical set-up was based on an earlier study [62] and is illustrated in Figure 1 and Figure 2.

We instructed the participants to navigate to a specific target item in the grid menu as fast and straight as possible. Hence, AFFINDU engaged the participants in a simple but still realistic HCI task. We told participants that AFFINDU reacted to their input. We implemented three different types of system reactions to this input:

1. Supportive adaptation: The system helps the participant to reach the target faster by rearranging the menu layout so that the number of remaining navigation steps is reduced. This kind of system adaptation is expected to induce a positive affective user reaction.
2. Impeding adaptation: The system hinders the participant reaching their target by rearranging the menu layout so that the number of remaining navigation steps is increased. In this condition, we assume induced negative affective user reactions.
3. No adaptation: As a baseline, the system does not perform any adaptive behavior.

The adaptation behavior was realized by calculating the Manhattan distance between the currently selected item and the target item [55]. Figure 2 illustrates examples of supportive and impeding system behaviors. In order to increase the ecological validity of the experiment, we provided a meaningful context and plausible explanations for the adaptive behavior of AFFINDU. We introduced the participants to the system with a cover story according to Figure 1. The behavior of AFFINDU was presented as a self-learning system. The participants were told that AFFINDU could be trained to learn the participants’ individual navigation strategies in a learning phase by monitoring participants during their navigation to several self-selected target items. In the learning phase, the participants could navigate freely and AFFINDU did not exhibit any assistance behavior. The subsequent testing phase then revealed how well the system actually “learned” the participant’s navigation style. Hence, participants believed that the self-initiated adaptations were based on their own training during the learning phase. The interaction with AFFINDU was realized on a controlled trial-based procedure as a visually cued task design including different task epochs (Figure 2). This allowed us to capture the participants’ affective processes during the navigation tasks by measuring power and functional connectivity responses in the monitoring task of the given adaptations of AFFINDU.

### 2.3. Experimental Design and Trial Procedure

All participants were comfortably seated upright in a chair in front of a computer screen equipped with a standardized keyboard lying on a desk and provided with instructions for the experimental design and cover story of AFFINDU. Their basic task was to navigate through the menu grid towards a defined target item. Participants were told that they could move from one item to the other by using the arrow keys and confirm the target item achievement by pressing the enter key.

Each participant completed a seventy-five-minute experimental session (see Figure 1) consisting of three blocks (A, B, and C). Each block consisted of a learning phase of five minutes and a testing phase of 20 min (navigation and monitoring part). In each testing phase, AFFINDU performed either supportive or impeding adaptations for 50% of the performed navigation steps, while for the other 50% no adaptations were performed. We ended up with (A) a positive block—including supportive assistance in combination with no assistance, (B) a negative block—including impeding assistance in combination with no assistance, and (C) a mixed block—including both supportive and impeding assistance in combination with no assistance. The adaptations were randomly distributed across each block. In total, participants were confronted with 30 supportive and 30 impeding adaptations: 20 supportive adaptations in block A, 20 impeding adaptations in block B and a mixed composition of ten supportive and ten impeding adaptations in block C. In order to reduce the impact of ordering, the blocks were randomized across participants. The block design enabled us to gain insights into the oscillatory EEG signatures of affect when participants are exposed to the system behavior for a longer period of time. Furthermore, it allowed us to evaluate the participants’ subjective affective experience of the interaction with AFFINDU after each block by using the Self-Assessment Manikin (SAM) in terms of valence, arousal and dominance [63].

Each trial was initiated by an instructional cue “navigate” that lasted for 2 s. This was followed by the navigation epoch requiring the participant to perform the navigation. The navigation epoch ended when pressing the enter button. It was followed by a 10 s resting epoch during which the menu disappeared and a black screen with a white fixation cross was displayed. Subsequently, the second instructional cue “observe” started lasting for another 2 s. It indicated that no keyboard input was required by the participant in the following replay-adaptation epoch. The replay-adaptation epoch lasted 5 s and consisted of two parts (Figure 2B,C): Within the first part, the move in the navigation path according to the participant’s keyboard input was replayed (replay navigation step). In the second part, the simulated assistance behavior was displayed, i.e., a supportive, impeding or no adaptation (system adaptation).

The two-epoch-design allowed us to capture the participants’ affective responses to adaptive rearrangements of the menu’s grid during the monitoring part of the system adaption epoch by measuring power and functional connectivity responses with EEG. The long-lasting resting epochs of 10 s were included due to other (neuro-) physiological recordings, i.e., functional near-infrared spectroscopy (fNIRS) that were applied in addition to EEG during the experiment but were not included in the present work. The complete experimental trial procedure and system behavior was presented in a web-based application in Google Chrome and programmed in JavaScript using the jQuery and Bootstrap libraries [55]. At the end of the experimental session, participants were debriefed, and all details of the procedure and cover story were revealed to them.

### 2.4. Measurement Set-Up and Data Recording

**Figure 3:**
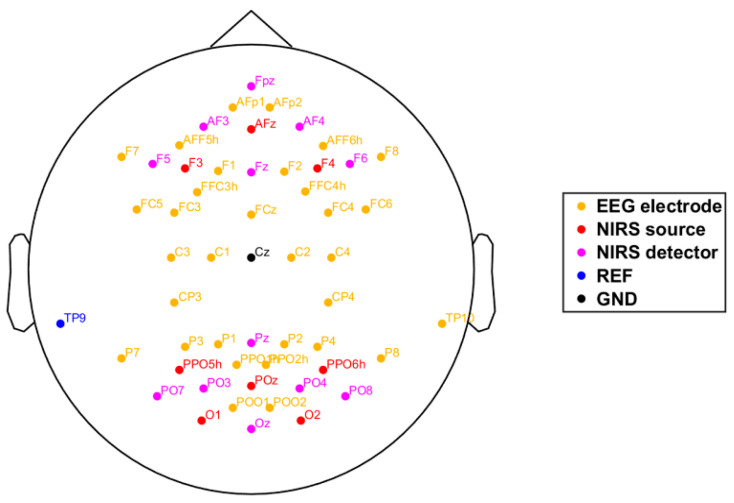
Two-dimensional (2-D) layout of the EEG electrodes and fNIRS optodes. Locations of the 32 EEG electrodes (orange); EEG ground (black) and reference position (blue), fNIRS sources (red), fNIRS detectors (pink).

Figure 3 illustrates the locations of the EEG channels and fNIRS optodes which were integrated in a standard EEG cap according to the extended international 10-05 system. Scalp EEG potentials were recorded (BrainAmp, Brain Products GmbH, Munich, Germany) from 32 positions with Ag/AgCl electrodes (actiCAP, Brain Products GmbH, Germany). The left mastoid was used as common reference and EEG was grounded to Cz. All impedances were kept below 20 kΩ at the onset of each session. EEG data was digitized at 1 kHz, high-pass filtered with a time constant of 10 sec and stored for off-line data analysis using the »BrainVision Recorder« Software (Brain Products, Munich, Germany). All EEG data analyses were performed with custom written or adapted scripts in MATLAB^®^.

### 2.5. Data Analysis of Subjective Affective Experience

Repeated-measures ANOVAs (rmANOVA) were carried out to analyze participants’ subjective affective evaluation of the adaptive system behavior in the three testing phase blocks. The three dimensions of the SAM (a) valence, (b) arousal, and (c) dominance were analyzed. The analysis included testing phase block (A—supportive system behavior, B—impeding system behavior, C—mixed system behavior) as a within-subject factor and was performed using SPSS (V20 IBM).

### 2.6. EEG Data Pre-Processing

For the further data analysis, the current study takes into account the EEG signals that were recorded during the testing phase of the three blocks.

In the first pre-processing step, all trials of the supportive and impeding system behavior from the three blocks were grouped together. Next, the EEG signals were detrended, zero-padded and re-referenced to mathematically linked mastoids [64]. All EEG signals were subsequently filtered using a band-pass filter between 0.5 to 48 Hz to calculate the event-related spectral perturbation (ERSP) and functional connectivity (FC) measures. The filtering procedure was performed with a first order zero-phase lag finite impulse response (FIR) filter.

For the analysis of ERSP and FC, the continuous EEG signals were split into stimulus-locked segments (replay-adaptation phase) of 6000 ms. Each segment consisted of (a) 2000 ms rest phase in which the fixation cross was presented ranging from −4000 to −2000 ms before stimulus-onset; (b) 2000 ms presenting the instructional cue »observe« ranging from −2000 to 0 ms before stimulus-onset, and 2000 ms of the replay-adaptation segment ranging from ms 0 to 2000 after stimulus-onset. The replay-adaption segment comprised of 1000 ms replaying the navigation step and the first 1000 ms of system adaptation. Segments were rejected when they contained a maximum deviation above 200 µV in any of the frontal EEG channels (AFp1, AFp2, AFF5h and AFF6h). We performed an independent component analysis (ICA) using the logistic infomax ICA algorithm as implemented in the EEGlab toolbox [65] on each un-rejected segments, and removed further cardiac, ocular movement and muscular artifacts. This was done by careful visual inspection of the topography, times course and power spectral intensity of the ICA components (mean ± SD of rejected components: 3.18 ± 0.91) [66,67].

### 2.7. Estimation of Event-Related Spectral Pertubation

**Figure 4:**
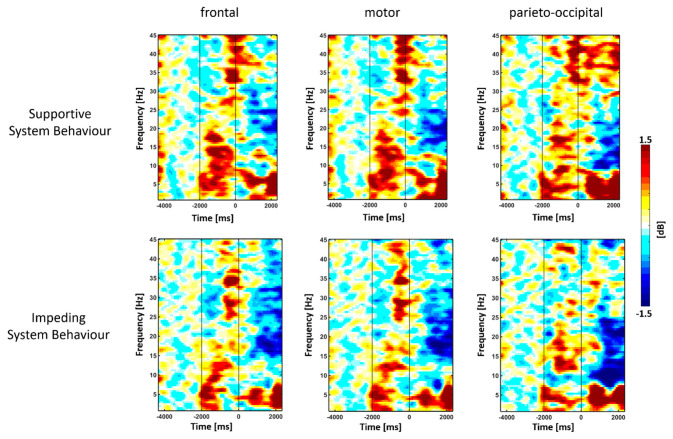
Grand-averaged time-frequency maps of the event-related spectral perturbations (ERSPs). The grand-averaged time-frequency of the ERSP to supportive (upper row) and impeding (lower row) system behavior averaged across different regions of interest. Electrodes overlying frontal regions (left column): AFp1, AFp2, AFF5h, AFF6h, F1, F2, F7, F8; motor related regions (middle column): FC5, FC3, FC6, FC4, FFC3h, FFC4h, C3, C1, C4, C2, CP3 and CP4; and parieto-occipital regions (right column): P8, P4, P7, P3, PPO2h, PPO1h, POO2, POO1. The ordinate represents the frequency axis, while the abscissa represents the time axis, with the rest epoch from−4000 ms to−2000 ms (first black line), the instruction epoch »observe« from−2000 ms to 0 ms (second black line), the replay navigation step from 0 to 1000 ms, and system adaptation from 1000 to 2000 ms. The time-frequency map is visualized on a decibel (dB) scale and normalized with respect to the rest epoch. Colors indicate event-related synchronization (ERS, red) and event-related desynchronization (ERD, blue).

To study regional oscillatory neuronal signatures, an event-related spectral perturbation (ERSP) time-frequency map, as implemented in the EEGLab toolbox [65] was calculated. This map was estimated between 1 and 45 Hz with a frequency resolution of 0.48 Hz across all time points and a time resolution of 33.33 ms for each channel, separately. For each participant, the ERSP was separately calculated trial-wise for all time points of the 6000 ms segments and averaged across trials of supportive and impeding system behavior. ERSP values have been normalized with respect to the rest epoch that precedes the instruction epoch »observe« and visualized across frequency between 1 to 45 Hz, and time with −4000 ms to −2000 ms comprising the rest phase, −2000 to 0 ms the instructional cue »observe«, 0 to 1000 ms the replay, and from 1000 to 2000 ms the system adaptation (see also Figure 4).

### 2.8. Estimation of Functional Cortical Networks

To study global oscillatory neuronal signatures, we further analyzed functional cortical networks. For the calculation of functional connectivity (FC), we utilized the imaginary part of the coherence function (iCOH) [68]. iCOH disregards relations at zero phase lag and is, therefore, insensitive to volume conduction properties. Thus, it indicates the relative coupling of phases, i.e., the time-lag between two brain processes. Since the original iCOH function might exhibit a spatial bias towards long-range synchronizations, we used the corrected version of the iCOH function (ciCOH) as suggested by [69]. ciCOH shares the same properties as the originally proposed version but includes additional features to compensate for the preference of remote interactions. For the analysis of FC, we focused on two temporal windows of equal size: (1) a baseline comprising the last second of the rest phase preceding the instructional cue »observe« and (2) the time window of interest (TOI) comprising the first second of the system adaptation phase likely to capture the immediate affective reaction.

The estimation of the ciCOH is based on the complex coherency function. Hence, cross-spectral densities were calculated from each valid segment (baseline and TOI) using a Fourier transformation of the EEG time series and spectrally smoothing the data according to a multi-tapering approach as implemented in the »Chronux« toolbox [70] (http://chronux.org/). A window size of 1000 ms with a step size of 100 ms was used. The 1000 ms segments were tapered using slepian functions, whereby the number of tapers applied was defined according to the equation 2*bandwidth-1 [71] with a bandwidth of 3, resulting in five tapers for spectral smoothing with a spectral bandwidth of δ*f* = 0.98 Hz. From the cross-spectra, the complex coherency function between channel pairs is defined as the normalized cross-spectrum for channels *i* and *j* (1):

(1) $${\mathrm{COH}}_{ij}\left(f\right)=\;\frac{S_{ij}\left(f\right)}{\sqrt{S_{ii}\left(f\right)S_{jj}\left(f\right)}}$$

where $S_{ij}\left(\cdot \right)$ is the cross-spectrum between channels *i* and *j*, and $S_{ii}\left(\cdot \right),\;S_{jj}\left(\cdot \right)$ represents the auto-spectra for channels *i* and *j*, respectively. From the complex coherency function the ciCOH is defined [69] (2)

(2) ciCOHij(f)= Im(COHij(f))(1−Re(COHij)2)

where Im(·) and Re(·) denote the imaginary and real part, respectively. The ciCOH was fisher z-transformed to fit a Gaussian distribution [68,72]. We evaluated the FC within three pre-defined frequencies of interest (FOI): (1) α-band (8–14 Hz), (2) β-band (15–35 Hz), and (3) γ-band (35–45 Hz).

We systematically evaluated the FC networks between the visual cortex (VIC) and the whole brain (all other EEG channels), for each FOI, by defining POO1 and POO2 (electrodes over the VIC) as the seed electrodes. In a next step, the FC measure was obtained by calculating the event-related ciCOH (ER-ciCOH) as the contrast between the TOI and baseline [68,73,74] (3):

(3) $$\mathrm{ER}-{\mathrm{ciCOH}}_{\mathrm{Seed},j}\left(\mathrm{FOI}\right)=\;\frac{\mathrm{abs}{\left({\mathrm{ciCOH}}_{\mathrm{Seed},j}\left(\mathrm{FOI}\right)\right)}_{\mathrm{TOI}\;}\;-\;\mathrm{abs}{\left({\mathrm{ciCOH}}_{\mathrm{Seed},j}\left(\mathrm{FOI}\right)\right)}_{\mathrm{Baseline}\;}}{\mathrm{abs}{\left({\mathrm{ciCOH}}_{\mathrm{Seed},j}\left(\mathrm{FOI}\right)\right)}_{\mathrm{Baseline}}}$$

where Seed denotes the seed electrode and abs indicates the absolute value of ciCOH. The ER-ciCOH is a measure of functional coupling between electrode sites with positive values indicating stronger functional connectivity during the TOI segment. While negative values indicate stronger coupling during the baseline segment. We obtained ER-ciCOH values for each trials of supportive and impeding system behavior, respectively, and averaged them for each participant separately.

### 2.9. Statistical Analysis of Event-Related Spectral Pertubations and Functional Connectivity

To analyze the impact of adaptive system behavior in the frequency domain (i.e., ERSP and FC), the neuronal signatures were compared between the supportive and impeding system behavior trials.

**Figure 5:**
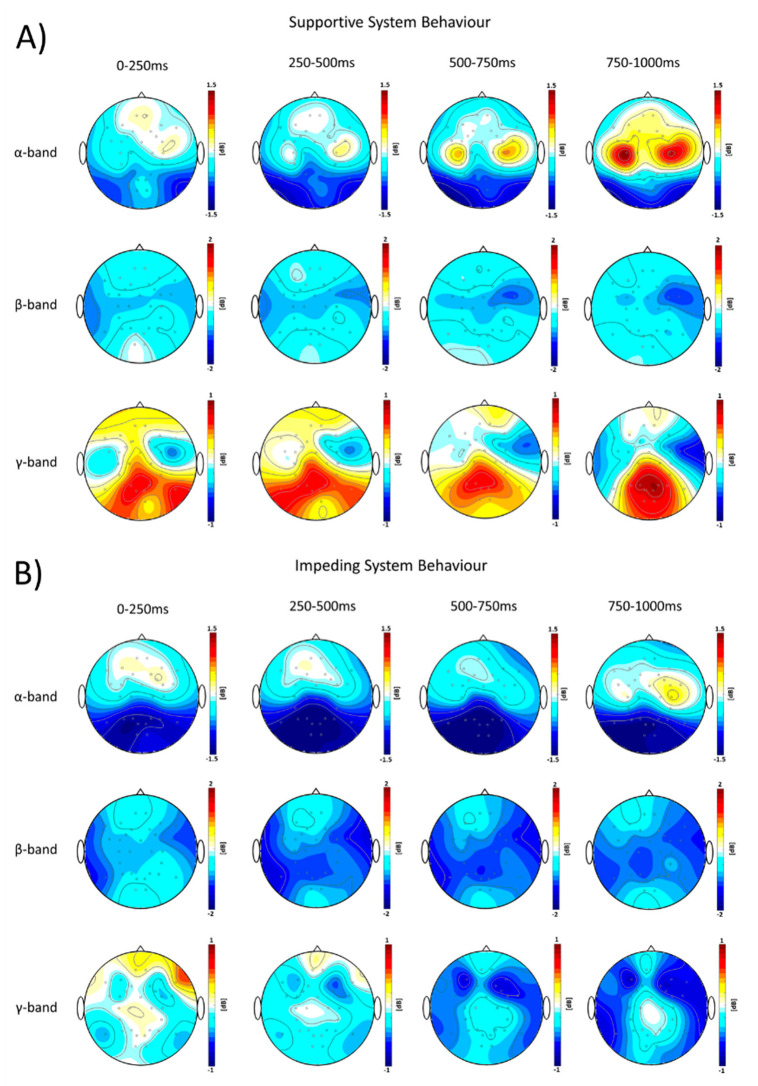
Grand-averaged topographical evolution of event-related spectral perturbations (ERSPs) to supportive and impeding system behavior. The plots show the evolution of the ERSP (visualized on decibel scale; dB) for the different frequency bands: α-band (8–14 Hz, first row), β-band (15–35 Hz, second row), and γ-band (35–45 Hz, third row) in four time intervals of the adaptation phase (0–250 ms: first column, 250–500 ms: second column, 500–750 ms: third column, and 750–1000 ms: fourth column) to supportive (**A**) and impeding system behavior (**B**). Colors indicate event-related synchronization (ERS, red) and event-related desynchronization (ERD, blue).

**Figure 6:**
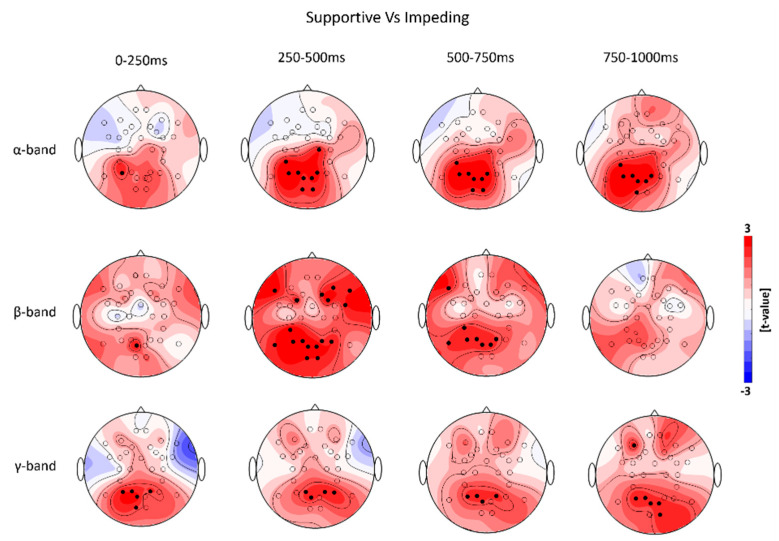
Evolution of regional neuronal signatures of affective reactions in the frequency-domain. The plots represent the evolution of *t*-value topography differences of event-related spectral perturbations (ERSPs) by comparing the supportive with the impeding system behavior trials. The oscillatory neuronal signatures were estimated for three different frequency bands: α-band (8–14 Hz, first row), β-band (15–35 Hz, second row), and γ-band (35–45 Hz, third row) in four time intervals of the adaptation phase (0–250 ms: first column, 250–500 ms: second column, 500–750 ms: third column, and 750–1000 ms: fourth column). Red color indicates higher modulations of ERSP (event-related desynchronization and event-related synchronization) during the supportive as compared to the impeding system behavior trials, while blue color indicates the opposite scenario. Electrode clusters, showing significant differences in the non-parametric randomization test, are indicated by filled black circles.

In order to evaluate regional oscillatory neuronal signatures in the frequency domain, the time-frequency maps of the ERPS for the supportive and impeding trials were divided into pre-defined FOI by calculating the mean values for the α-band (8–14 Hz), β-band (15–35 Hz), and γ-band (35–45 Hz) in four different time intervals (0–250, 250–500, 500–750, and 750–1000 ms after the onset of the system adaptation) (see also Figure 5 and Figure 6).

We applied a multiple dependent samples *t*-test (supportive versus impeding system behavior trials) on the level of individual electrodes for the ERSP and ER-ciCOH. For this purpose we used a cluster-based non-parametric randomization approach which included correction for multiple comparisons [75,76] as implemented in the »FieldTrip« toolbox [77] (http://fieldtrip.fcdonders.nl/). Here, a dependent sample *t*-statistic was conducted to establish the topography of neuronal signatures in which the ERSP (for each FOIs and time interval) and ER-ciCOH (for each FOIs) revealed significant differences between the supportive versus impeding system behavior conditions. Hence, *t*-values exceeding the a priori threshold of *p* < 0.05 (uncorrected) were spatially clustered based on neighboring electrodes. The cluster level statistics were defined as the sum of *t*-values within every cluster. The correction of multiple comparisons was realized by calculating the 95th percentile (two-tailed) of the maximum values of summed *t*-values estimated from an empirical reference distribution. *t*-values exceeding this threshold were considered as significant at *p* < 0.05 (corrected). The reference distribution of maximum values was obtained by means of a permutation test (randomly permuting the ERSP and ER-ciCOH across the supportive and impeding system behavior conditions for 1000 times).

## 3. Results

### 3.1. Subjective Affective Reations to Adaptive System Behavior

The rmANOVAs revealed the main effects of the testing phase block on the valence, arousal and dominance dimensions. Mauchly’s test of sphericity showed that the assumption of sphericity had been violated for the valence, arousal and dominance dimensions, *χ*^2^ (2) = 8.15, *p* = 0.017, *χ*^2^ (2) = 15.63, *p* ≤ 0.001, *χ*^2^ (2) = 11.62,00, *p* = 0.003, respectively. Based on their epsilon value the rmANOVAs for valence and arousal were corrected using the Greenhouse–Geisser method, while for dominance the Huynh–Feldt correction was used.

The rmANOVA showed a significant main effect of the testing phase block on the ratings of the valence dimension, *F*(1.37,19.10) = 10.70; *p* = 0.002. Post-hoc pairwise comparisons via a two-sided *t*-test revealed that the testing phase block A (*M =* 6.07, *SD* = 0.42) comprising supportive system behavior was rated significantly more positive than the testing phase block C (*M* = 4.80, *SD* = 0.31; A vs. C, *p* = 0.028) and testing phase block B (*M* = 3.80, *SD* = 0.47; A vs. B, *p* = 0.009). No statistically significant difference was observed between the testing phase blocks C and B. However, we found a main effect of testing phase block on the ratings of the arousal dimension, *F*(1.45,49.37) = 5.95; *p* = 0.01. Post-hoc pairwise comparisons indicated that arousal ratings were generally moderate for all testing phase blocks. Still, the testing phase block B (*M* = 4.83, *SD* = 0.39) was rated significantly higher than the testing phase block A (*M* = 3.49, *SD* = 0.33; B versus A, *p* = 0.03). No statistically significant difference between testing phase blocks C and B was found. Regarding the dominance dimension, there was a statistically significant main effect of testing block *F*(1.60,54.44) = 4.45, *p* = 0.023, indicating a trend of a decrease in rating scores from block A over block B to block C. However, none of the results of the post-hoc pairwise comparisons reached significance.

### 3.2. Regional Frequency Domain Specific Neuronal Signatures of Affective Processes to Adaptive System Behavior

Figure 4 represents the time-frequency map of event-related spectral perturbations (ERSP) as the grand-average over trials and participants, separately, for supportive (Figure 4, upper row) and impeding system behavior (Figure 4, lower row). The dynamics of ERSP showed a difference of oscillatory power modulations of event-related desynchronization (ERD) in the α-band and β-band, and event-related synchronization (ERS) in the γ-band among frontal regions, motor-related regions, and parieto-occipital regions between the two system behavior conditions during the adaptation time window. The α- and β-band ERD tend to be stronger for the impeding system behavior (Figure 5B), while the γ-band ERS was increased after supportive system behavior (Figure 5A).

The non-parametric randomization test revealed significant changes of α- and β-band ERD and γ-band ERS, during the respective four time-windows after the onset of system adaptation (Figure 6).

More specifically, we observed stronger α-ERD starting at 250 ms after the onset of system adaptation in bi-lateral parieto-occipital regions when participants observed impeding system behavior (Figure 6, upper row). Furthermore, stronger β-ERD starting at 250 ms after the onset was found in bi-lateral fronto-central and parieto-occipital regions while the frontal activity decreased in the successive interval from 500–750 ms. Parieto-occipital regions displayed significantly stronger β-ERD even 500–750 ms after onset of the system adaptation. However, the β-ERD was still significantly stronger for the impeding system behavior condition (Figure 6, middle row).

For the γ-band ERS the non-parametric randomization test revealed significant differences at bilateral parieto-occipital regions compared to the baseline starting at the shortest latencies (0–250 ms) after onset of the system adaptation. The γ-band ERS was stronger when participants observed supportive system behavior (Figure 6, lower row).

### 3.3. Global Frequency Domain Specific Neuronal Signatures of Affective Processes to Adaptive System Behavior

**Figure 7:**
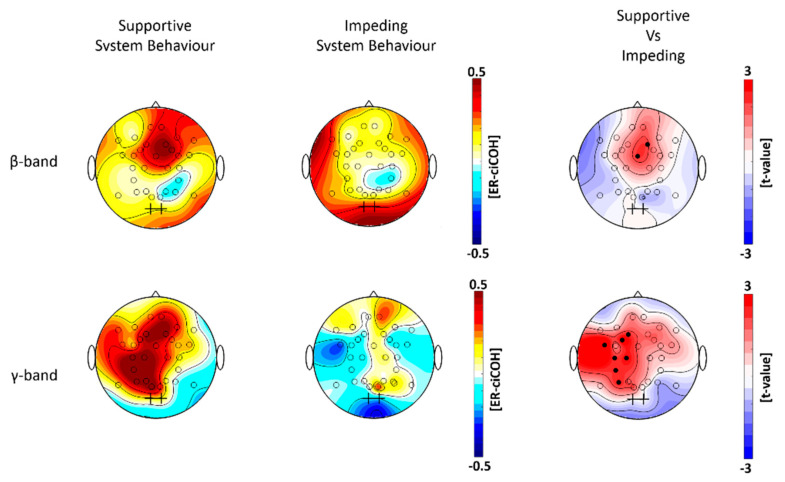
Global neuronal signatures of affective reactions in the frequency-domain. The plots represent the cortical modulations of the functional connectivity for the β-band (15–35 Hz, upper row), γ-band (35–45 Hz, lower row). The plots on the left and middle column show the grand-averaged event-related corrected imaginary coherence (ER-ciCOH) for the supportive (left column) and impeding (middle column) system behavior trials. Colors indicate increased functional coupling (red) and decreased functional coupling (blue) within the time window of interest (one second after system adaptation) relative to baseline segment. The plots on the right column show the *t*-value topographies of the ER-ciCOH as a contrast between supportive versus impeding system behavior trials for the β-band (upper) and γ-band (lower). The black crosses in all plots indicate the seed electrodes position (POO1 and POO2). Electrode clusters, showing significant differences in the non-parametric randomization test, are indicated by filled black circles. Red color indicates higher modulations of ER-ciCOH during the supportive as compared to the impeding system behavior trials, while blue color indicates the opposite scenario.

The non-parametric randomization test revealed significant changes of ER-ciOCH for the β-band and γ-band (Figure 7, right column), but not for the α-band.

We observed higher functional connectivity in the β-and γ-band during the TOI (one second after the onset of system adaptation) compared to the baseline segment for the supportive system behavior (Figure 7, left column). For impeding system behavior, functional connectivity increased only in the β-band, while functional coupling was even stronger during the baseline compared to the TOI in the γ-band (Figure 7, middle column). More specifically, we observed that the seed electrodes overlying the visual cortex showed stronger functional connectivity with electrodes overlying the medial prefrontal regions in the β-band when participants observed trials with supportive system behavior (Figure 7, right column, upper plot). For the γ-band, the seed electrodes overlying the visual cortex showed stronger functional connectivity with electrodes overlying the left fronto-central and parietal regions in the supportive system behavior condition (Figure 7, right column, lower plot).

## 4. Discussion

The current study investigated oscillatory neuronal signatures in the electroencephalography (EEG) that represent affective processes of participants during the interaction with an adaptive computer system. To investigate affective user reactions in an ecologically valid and close-to-naturalistic approach, we used a validated experimental paradigm called AFFINDU [55]. AFFINDU allows us to systematically manipulate the participants’ affective states by adaptive system behaviors that either support or impede their goal achievement during a navigation task. In the study presented here, the participants’ subjective ratings showed that supportive adaptations were evaluated significantly more positive and less arousing than impeding adaptations of the system. Our results further indicate that the cover story worked effectively, and the participants believed that they were interacting with an actual adaptive system.

### 4.1. Difference in Regional and Global Oscillatory Neuronal Signatures

The simultaneous EEG recordings during the HCI allowed us to gain new insights into the functional basis of affective processing during the interaction with assistive technology. We observed regional and global oscillatory neuronal oscillatory signatures by studying event-related spectral perturbations (α- and β-band ERD and γ-ERS) and functional connectivity in the β- and γ-band during the interaction. These neuronal signatures seem to be sensitive to positive and negative affective reactions to adaptive system behavior, respectively (Figure 6 and Figure 7). Measures of the oscillatory activity in circumscribed cortical regions as well as the interrelations among certain regions based on frequency-specific neuronal correlations in large-scale cortical networks have been proposed to serve as spectral neuronal signatures underlying learning as well as most cognitive, motor and emotional functions [78,79,80]. Event-related spectral perturbations in different frequencies display certain neurophysiological properties within circumscribed cortical regions, indexing sensory, higher order cognitive and motor processing during different stages of task-related processing. These activities are commonly summarized as event-related synchronization/desynchronization (ERD/ERS) [81]. ERD/ERS represents oscillatory power changes before, during or after a stimulus, reflecting changes in firing properties of neuronal cell assemblies in cortical regions underlying EEG electrodes [81]. We observed stronger γ-band ERS in electrodes overlying parieto-occipital regions during the observation of supportive system behavior, while impeding system behavior induced a decrease in γ-band oscillatory power with simultaneously higher α- and β-band ERD. Previous studies suggest that the α- and β-band ERD represent electrophysiological correlates of activation in cortical regions that are involved in sensory and higher order cognitive information processing and the production of motor behavior [82,83,84,85,86]. Hence, a visual input results in desynchronization of occipital α- and β-band rhythms and simultaneous increases in γ-band synchronization across parietal and occipital cortical regions. This signature indicates visual perception and task-relevant categorization [84,87,88,89,90,91]. In contrast to α- and β-band ERD, γ-band ERS represents an electrophysiological correlate of the enhancement of cooperative and synchronized behavior in larger number of neurons underlying EEG electrodes. This synchronization is important for a rapid active binding of sensory information in visual regions [92].

Our results show a higher α-band ERD at electrodes overlying centro-parietal and parieto-occipital regions during impeding system behavior (Figure 6, upper row), potentially indicating a higher reactivity of the motor cortical network. Moreover, we observed significantly higher β-band ERDs in electrodes overlying bi-lateral fronto-central and centro-parietal regions at the time interval starting at 250 until 750 ms after the onset of the AFFINDU adaptation (Figure 6, middle row). These topographical activation maps of α- and β-ERD might represent an initial preparatory up-regulation of the motor cortical network and mental effort for the possible initiation of a movement during the observation of impeding system behavior [84,86,93], since the presented AFFINDU behavior is in high conflict with the goal achievement of the participant. This interpretation is further supported by the observation of a stronger α-ERS at bilateral central regions during supportive trials in the last time window from 750–1000 ms (Figure 5). Previous studies associate correlates of α-ERS with an active inhibition of the motor cortex [94]. Therefore, our finding could indicate an active inhibition of motor related movement intentions reflecting no conflict with planned actions of the participants during supportive system behavior. Conversely, the impeding adaptive system behavior is not consistent with the goal-achievement and motivation of the participants to complete the assigned navigation task. Thus, the stronger α- and β-band ERD during impeding trials might point to a higher readiness for the recruitment of other cortical resources potentially associated with mental imagery, error correction and increased alertness [81].

For α- and β-band ERD, a valence-related role has been described [95,96,97,98,99], showing the general tendency of higher activity towards negative visual emotional stimuli compared to positive and neutral ones. Previous studies demonstrated that both, pleasant and unpleasant stimuli, elicit γ-band increases in subcortical and cortical regions [45,49,100,101,102,103]. These studies showed a general tendency for neuronal optimization during rapid processing of emotionally relevant stimuli (positive and negative) compared to neutral stimuli. Increased up-regulation of neuronal oscillatory activity has further been described for γ-band activity among distributed cortical regions, i.e., in visual, prefrontal, parietal and posterior cortices for emotional stimuli relative to neutral stimuli [101]. In addition, increases in the γ-band at short latencies after the stimulus onset were found to reflect the conscious perception and awareness of emotionally relevant contents, which is important for rapid discrimination of aversive stimuli [101,104]. Taken together, our results are in line with previous findings and provide evidence for the link between γ-frequencies and bottom-up related processing of sensory information in the visual system [105]. During the AFFINDU experiment, the neuronal activation in γ-frequencies seemed to provide information whether the presented system adaptation was meaningful or not.

Functional processing of emotional stimuli from the environment first arrives at thalamic nuclei, and is then processed via two main pathways consisting of (1) subcortical and (2) cortical neuronal circuits [106,107]. Within these neuronal circuits, the information is directed towards sensory and parietal cortices. Thus, when the information is processed in the visual system, there might be a pronounced bottom-up processing of information for supportive behavior as indicated by stronger oscillatory γ-band ERS [105]. In addition, the neuronal processes are re-allocated to change intrinsic strategies during impeding system behavior as indicated in α- and β-band ERD [80,84]. Taken together the regional oscillatory neuronal activity found in our study indicates ongoing processes of affective regulation during the observation of the adaptive behavior by the participant. The spectral power in the γ-band appears to be a neuronal signature associated with the observation of the expected system behavior. While, the α- and β-band activity might indicate a higher regional activity to recruit further cortical resources associated with the observation of the impeding system behavior probably evaluated as inappropriate. Thus, our results show that frequencies in the α-, β-band and γ-band are sensitive for different stages of affective stimulus processing during the interaction with an adaptive computer system.

Attention-related effects of neuronal synchronization in the γ-band between functionally connected the visual, parietal and prefrontal cortices have previously been described during visuospatial tasks [90]. The functional connectivity profiles in our study are in accordance with recent findings showing activation of attention-related networks including parietal and prefrontal regions recruited by the visual system when processing emotional stimuli [46,47]. Synchronized activity between prefrontal and parietal regions in the γ-band is known to be involved in attentional and motor control processes [108]. A motivational role has recently been reported during emotional processing associated with an activation of the fronto-parietal network in the γ-band [109]. This network activity shows a preponderance towards the left hemisphere that is important for the active self-regulation of affective reactions [110]. Attention-related mechanisms during the processing of emotional situations may, therefore, be driven by low-level stimulus properties, e.g., perceptually salient exogenous stimuli. However, they could also be related to the volitional goals and endogenous goal-directed behavior of the person himself or herself [111]. Within this network, the role of the visual cortex may act to optimize early perception of specific features indicating emotional relevance for the participants [104]. The role of the parietal cortex is crucial in representing associations between the cognitive control circuits in the prefrontal cortices [100] and motivation-oriented plans with the purpose of flexible adaptation and re-allocation of neuronal process to current task demands [112]. The prefrontal cortex (PFC) is important for higher-order attention and cognitive control to enable inhibition of task-irrelevant information and the prioritized processing of task-relevant information [106,109,113,114]. The seed electrodes overlying visual regions functionally connected with electrodes over the midline prefrontal in the β-band further support this argument of the top-down related role of the PFC. This top-down related PFC activation seems to be important for monitoring the presented system behavior and to dissociate supportive from impeding trials.

From a methodological point of view, the present study has certain limitations regarding the localization of functional coupling among distant EEG sensors and possible volume conduction effects in the functional connectivity analysis [115,116,117]. The imaginary part of the coherency function (iCOH) is a connectivity measure insensitive to volume conduction properties ignoring relations at zero (or close to zero) time lag [69,118]. It is important to note that the classical iCOH might exhibit a spatial bias towards long-range interactions, thus, possibly underestimating short-range interactions at the sensor level. The corrected form of the iCOH function (ciCOH), which is used in the present study, intends to address this bias by maximizing the imaginary part of the cross-spectrum [69,118]. Thus, the ciCOH function improves the spatial specificity further when connectivity is studied on the sensor level, which potentially leads to observations of interactions otherwise hidden in the noise [69,118]. The field spread problem of neuronal signals recorded at scalp EEG electrodes is a serious problem when the anatomical relationship of cortical structures is of interest. One possible way of improving the spatial specificity and further reducing the possible volume conduction biases is the use of source reconstruction, which in turn introduce other downsides such as the inverse problem and source leakage [115,116,117]. Further studies could investigate the functional connectivity profiles in combination with inverse source modelling analysis.

### 4.2. Processing of Computer-Generated Feedback

The AFFINDU experimental paradigm was designed to research affective processes towards an automated system showing either impeding or supportive behavior in an observational navigation task. One main motivation to choose this experimental paradigm was (a) its suitability in a closed human–computer interaction loop and (b) the interactive nature of the task itself. The participants first provided their own navigation input and observed the system-initiated behavior to the given input afterwards. Thus, the system-initiated behavior could also be interpreted as a feedback to the suggested navigation provided by the participants. Since the system-initiated behavior was either congruent or incongruent with the participants’ goals, the system feedback could be perceived either positively or negatively depending whether expectations were violated or not. Sheridan and Parasuraman [119] defined five functions of a human supervisor within the interaction with an automated system: (1) plan off-line, (2) teach the automation, (3) monitor the automation’s execution of the plan, (4) intervene to abort or assume control as necessary, and (5) learn from experience. Within the AFFINDU experiment, participants experienced the first three functions during each interaction trial with the system. Since we did not provide the possibility to execute the fourth function of intervention, participants could not correct impeding system behavior in trials preventing goal achievement. However, we observed regional oscillatory and global functional coupling which might hint towards the motivation and intention to assume control due to inappropriate observed system behavior. Recent studies investigated neuronal correlates associated with socio-emotional language processing of a self-related feedback either provided by a human or randomly generated by a computer [120,121]. Their results revealed that the same not only positive but also negative feedback perceived by random computer-generated feedback elicit less pronounced event-related potentials (ERPs) compared to ERPs elicit by feedback from humans. The authors concluded that the perception and processing of positive and negative feedback changes quantitatively and qualitatively when received in a human–computer interaction due to the missing social context. However, they observed larger late positive potentials in the positive feedback condition compared to the negative feedback condition not only in a social context but also in the interaction with a computer. This so-called self-positivity bias could be an explanation for the high subjective valence and dominance ratings for the supportive system behavior in our AFFINDU experiment. Since participants provided their intended navigation and taught (based on the cover story) the system in the learning phase, they might attribute the goal achievement in the supportive system behavior of AFFINDU to their initial training of the system. Due to the repeated interaction with the system showing not only supportive but also impeding behavior, it is important to mention that the concept of trust might play a mediating role in the perception and evaluation. Trust is defined as the attitude that an agent or system will support to achieve a pursued goal [122,123]. Previous research has provided evidence that trust in an agent or system is strongly influenced by its reliability in task performance and negatively correlated with perceived errors of the automated system [124,125,126]. Since we did not measure the level of trust evaluated by the participants and its time course within the repeated interaction comprising supportive but also impeding trials, the neurophysiological signatures might be influenced by this mediating factor. Further studies could investigate the possible effects of instable task performance, trust evaluation and their neurophysiological correlates.

## 5. Conclusions

The present results provide important new insights into regional and global oscillatory EEG activity in cortical processing as it is reflected in the ERD/ERS and functional connectivity modulations to supportive and impeding assistance during the interaction with adaptive systems. Our results revealed significantly higher α-band and β-band ERD in centro-parietal (with a left hemispheric preponderance) and parieto-occipital regions as well as early β-band ERD at bi-hemispheric fronto-central regions during impeding system behavior. Supportive system behavior activated significantly higher γ-band ERS in bi-hemispheric parietal-occipital regions. Furthermore, the supportive system behavior modulated significantly higher functional coupling of remote β-band and γ-band activity in the medial frontal and left fronto-central and parietal regions, respectively. A better understanding of the oscillatory signatures associated with affective processes in a close-to-naturalistic interaction with an adaptive system is a precondition for the development of EEG-based neuroadaptive assistance loops. In a next step, we aim at exploring the suitability of the identified oscillatory signatures as features (and in combination with other possible EEG-based features) in various machine learning algorithms for classifying affective states during the HCI. Furthermore, we plan to repeat this study with a higher spatial sampling of EEG electrodes (>64 channels) to perform the connectivity analysis in the source space in order to gain a deeper understanding of the underlying sources in the functional connectivity. This will allow us to integrate recognized affective user states as additional feedback into adaptive systems so that they can appropriately optimize their behavior towards increased safety and user acceptance.

## Data Availability

The dataset presented in this study is available for qualified researchers upon reasonable request from the corresponding author. The dataset is not publicly available since the consent of the participants was not obtained.
